# Virulence Attributes of Low-Virulence Organisms

**DOI:** 10.1155/S1064744994000463

**Published:** 1994

**Authors:** Bryan Larsen

**Affiliations:** Department of Obstetrics and Gynecology Marshall University School of Medicine 1801 6th Avenue Huntington WV 25703 USA

## Abstract

The vast majority of infections involving female pelvic structures arise from organisms that are members of the normal flora. In addition, exogenous organisms that invade through the lower genital tract must interact with organisms that are part of the host's flora. In contrast to the concept that the normal flora is entirely innocuous, recent research has begun to identify what appear to be virulence attributes among these ordinarily low-virulence organisms. Most of our understanding of virulence has been derived from highly virulent organisms, of which *Neisseria gonorrhoeae* provides an example of relevance to the female genital tract. A review of the virulence factors of the gonococcus is presented to serve as an example of the variety of virulence properties associated with pathogenic bacteria. Molecular biology has begun to clarify one of the important paradigms of pathogenic bacteriology—that bacteria change their expression of virulence properties in response to their location within a host or to the stage of infection. Thus, infection involves  not only the possession of virulence factors, but also the carefully controlled use of those factors. Virulence is often controlled by the coordinate expression of many virulence-associated genes in response to one environmental signal. With regard to low- virulence organisms present in the female lower genital tract, we are beginning to identify some of their virulence attributes. Examples from the work of our laboratory include the hemolysin of *Gardnerella vaginalis* and an immunosuppressive mycotoxin produced by *Candida albicans*. Demonstrating the coordinate expression (or other control mechanisms) of virulence factors in these sometimes innocuous and sometimes inimical organisms represents the next frontier in the study of normal vaginal microbiology.


Infectious Diseases in Obstetrics and Gynecology 2:95–104 (1994)
© 1994 Wiley-Liss, Inc.

### Virulence Attributes of Low-Virulence Organisms

**Bryan Larsen**
*Department of Obstetrics and Gynecology, Marshall University School of Medicine, Huntington, WV*

ABSTRACT

**The vast majority of infections involving female pelvic structures arise from organisms that are members of the normal flora. In addition, exogenous organisms that invade through the lower genital tract must interact with organisms that are part of the host's flora. In contrast to the concept that the normal flora is entirely innocuous, recent research has begun to identify what appear to be virulence attributes among these ordinarily low-virulence organisms. Most of our understanding of virulence has been derived from highly virulent organisms, of which *Neisseria gonorrhoeae* provides an example of relevance to the female genital tract. A review of the virulence factors of the gonococcus is presented to serve as an example of the variety of virulence properties associated with pathogenic bacteria. Molecular biology has begun to clarify one of the important paradigms of pathogenic bacteriology—that bacteria change their expression of virulence properties in response to their location within a host or to the stage of infection. Thus, infection involves not only the possession of virulence factors, but also the carefully controlled use of those factors. Virulence is often controlled by the coordinate expression of many virulence-associated genes in response to one environmental signal. With regard to low-virulence organisms present in the female lower genital tract, we are beginning to identify some of their virulence attributes. Examples from the work of our laboratory include the hemolysin of *Gardnerella vaginalis* and an immunosuppressive mycotoxin produced by *Candida albicans*. Demonstrating the coordinate expression (or other control mechanisms) of virulence factors in these sometimes innocuous and sometimes inimical organisms represents the next frontier in the study of normal vaginal microbiology.** © 1994 Wiley-Liss, Inc.

KEY WORDS
Female genital tract, pathogenic bacteria, gonococcus, infection, vaginal flora

A virtual avalanche of data providing an understanding of virulence at cellular and molecular levels has occurred in the last decade, largely as a result of the availability of laboratory methods that permit the investigation of gene expression and regulation. However, the majority of basic work done on virulence has focused, not surprisingly, on the seemingly most dangerous microorganisms. For example, the organism responsible for plague (*Yersinia pestis*) and its near kin (*Y. enterocolitica*) have been studied extensively, resulting in the recognition of more than 2 dozen gene products that either function directly as virulence factors or are involved in the regulation of virulence.[1] Likewise, organisms that generally appear in association with specific disease entities (anthrax, tuberculosis, toxic shock syndrome, salmonellosis) have also been studied to the level of molecular detail. However, many opportunistic organisms, sometimes described as low-virulence organisms, have received less scrutiny.

Despite our burgeoning understanding of virulence attributes and the genes responsible for them, the basic conceptual framework for describing the development of infectious diseases on the level of the entire host can still be broadly defined in terms of the virulence and number of microorganisms counterpoised by the defense mechanisms of the

**Address correspondence/reprint requests to Dr. Bryan Larsen, Department of Obstetrics and Gynecology, Marshall University School of Medicine, 1801 6th Avenue, Huntington, WV 25703.**

Clinical Study

Received February 9, 1994
Accepted June 13, 1994

host. What has come about in the half century since Theobald Smith[2] formulated the concept of infectious disease in these terms is the identification of the actual molecular components of both virulence and host defense as well as a recognition of the dynamic interaction of these factors.

Historically, heroic efforts were required to convince skeptics that living things too small to be seen with the naked eye could cause devastating diseases affecting a host many billions times larger than the microorganism. In the golden age of microbiology, such pioneers as Koch and Pasteur identified different species of microorganisms and associated each one with certain typical infectious processes. The differences among species were consistent as were the diseases the organisms caused, giving rise to the concept, "one microorganism—one disease." There was little suspicion at that time that a single species of microbe may behave differently or in a more virulent manner, depending on its environment. Today, we recognize that microorganisms have the ability to regulate their virulence properties in response to their surroundings. Therefore, it is not only necessary to identify the microbial factors that confer virulence on the organism, but equally important to discover the environmental signals regulating the production of these factors. With regard to those organisms displaying little intrinsic virulence, the state-of-the-art still lies in identifying virulence attributes, while the task of identifying the genetic regulatory mechanisms remains to be investigated. In this article, some examples are provided to illustrate the developing science surrounding the virulence of microorganisms found in the female lower genital tract, organisms typically deemed to exhibit minimal virulence.

### VIRULENCE AND HOST DEFENSE

The dynamic relationship between host defense and microbial virulence in infectious diseases indicates that it is insufficient to view virulence attributes as a collection of substances that do some harm to host cells. Microorganisms may enhance their virulence by producing factors that act against host defense factors or in response to them. Some instructive examples may be found with *Neisseria gonorrhoeae,* which possesses virulence factors that specifically circumvent host defenses.

### Gonococcal Pilin

As with any invasive organism, the host responds to infection by the production of an antibody response. Each bacterial pathogen may present a very large number of antigenic epitopes to which antibody may be produced. When the immune response blocks the function of a key virulence factor, the infectivity of the microorganism should be severely limited. However, antigenic variation can, in effect, make such a virulence factor a moving target, leaving the immune system prepared to respond to an antigenic display that is passé.[3] The gonococcal pilus is considered to be one of the most significant virulence factors because it represents the adhesive surface component allowing attachment of the microorganism to columnar epithelial cells. This represents the initial step in the natural history of gonococcal disease. The gonococcus is able to undergo phase variation (at low frequency) whereby the organism becomes pilus negative, a condition resulting in loss of infectivity. Antigenic variation (which occurs with relatively high frequency) results in alterations of the primary structure of the pilin molecule, the approximately 18 KD subunit forming the pilus assembly. Both antigenic and phase variations occur as a result of rearrangement of the genetic material involved in pilin synthesis, expression, and transport to the microorganism's exterior. Two mechanisms are responsible for antigenic and phase variations: intragenic recombination within the chromosome of the gonococcus or intergenic recombination with transforming DNA from another gonococcal cell.

Genetic studies[4] of pilus production have been reviewed recently, and an abbreviated summary of the pilus gene rearrangements is noted here. The pilus gene is composed of expressed (*pilE*) and silent (*pilS*) loci. The silent region lacks a promoter and is composed of numerous copies of the pilin coding sequence, but these are truncated to varying degrees. Thus, while they do not encode the pilin produced by the gonococcus, they provide a source of genetic material from which the organism can generate antigenically diverse pilin proteins. The expressed locus produces a prepilin which must be post-translationally modified to replace an N-terminal export signal sequence with N-methylation of the initial phenylalanine of the pilin. Although the pilin molecule contains relatively conserved regions at the N-terminus of the molecule, there are 6 "minicassettes" located from the middle to the C-terminus of the pilin molecule that typically undergo recombination with material from the *pilS* locus, resulting in pili displaying new antigenic epitopes.

Although reassortment of the variable regions of the pilin gene results in antigenic variation, phase variation occurs when recombination leads to a pilin that is post-transcriptionally modified at amino-acid number 40 instead of the normal cleavage of the signal peptide at position 1. This type of pilin cannot be assembled into fimbrial structures and is either secreted in soluble form or accumulates in the periplasmic space as very long pilin molecules.

Thus, while the gonococcal pili represent a virulence attribute, pilin antigenic variability represents a further enhancement of their virulence by frustrating the immune response.

### IgA1 Protease

Proteases of various kinds with a myriad of specificities have been implicated as virulence factors among microorganisms. Simultaneously, it must be noted that the host also employs proteolytic enzymes as part of its defense against microbial pathogens.

One class of bacterial proteases which has proved especially intriguing for investigators are proteases that use human IgA1 as a substrate.[5] IgA proteases are usually associated with microorganisms that invade the host from mucosal sites. These include the gonococcus, meningococcus, *Streptococcus pneumoniae, S. sanguis,* and *Hemophilus influenzae.* The enzyme in the gonococcus is produced as a large molecule which is cleaved to produce the protease and subunits that provide for secretion through the gonococcal membrane to the extracellular environment.[6] The enzyme cleaves the antibody molecule in the vicinity of the hinge region, releasing the Fab fragment, an event sometimes whimsically referred to as "fabulation." Intuition would dictate that such a system functions as the gonococcal answer to secretory immunity, but we do not yet have definitive proof of the role of IgA1 protease as a virulence attribute. Much remains to be learned about the concentration and specificity of antibody that comes in contact with mucosal pathogens and how secretory antibody may adversely affect the gonococcus. Likewise, much remains to be learned about the in vivo concentration and efficacy of the gonococcal protease and whether it actually renders IgA ineffectual at the site of infection.

### Gonococcal Iron Acquisition

Iron has long been recognized as having a role in microbial virulence. Although it is a critical nutrient for all organisms, a considerable number of older studies indicated[7] that microorganisms injected into experimental animals in the presence of blood or heme produced infections more aggressive than those produced by a similar inoculum injected in the absence of excess iron. Many bacteria obtain iron by synthesizing chelators (siderophores) which have the ability to acquire iron from the host. However, *N. gonorrhoeae* is not a siderophore producer and therefore must use an alternative system for iron acquisition. The gonococcus produces specific receptors for transferrin and lactoferrin, the iron binding/transporting proteins of the host.[8] The organism does not conduct a robbery of the iron, but more like a hijacking, obtains iron by appropriating the vehicle along with its load of iron. Recent evidence indicates that the transferrin receptor system is a multicomponent system containing 2 proteins and showing some antigenic variability.[9] Although there is immunologic cross-reactivity between transferrin ligands found in gonococci, meningococci, and *H. influenzae,* the bacterial product does not cross-react with human transferrin receptors.

It should be emphasized that the operation of this iron-acquisition system is a regulated one. In the presence of iron excess, special iron-acquiring systems are not needed. Therefore, iron serves as a regulatory signal for a group of bacterial products collectively referred to as FeRPs (iron-repressible proteins). Other regulatory systems have been noted as well in the gonococcus. In addition to the *pilE* locus, the operon contains the *pilA* and *pilB* loci which appear to have regulatory functions over outer-membrane proteins which, in turn, have an influence on virulence, cell invasion, and serum resistance. These loci appear to be responsive to environmental signals and have homology with regulatory elements of other bacterial systems.

### VIRULENCE PARADIGM

The reason that microorganisms control expression of virulence factors is probably more complex than simply a matter of conserving metabolic energy when some factor is not needed. Many pathogenic organisms have exceedingly complicated lifestyles, such as those involved in zoonotic diseases. An organism like the plague bacillus may have to adapt to such diverse environments as a warm- or cold-blooded host and different species of mammals. Only by delicate regulation of growth and virulence factors can the organism be successful.

At first glance, the life of the gonococcus seems simple in contrast to an organism like the plague bacillus. However, while its normal environment is within the human host, the gonococcus inhabits a variety of locations in that host (cervix, rectum, urethra, bloodstream, fallopian tubes, and other sites) and each of these sites has a particular tissue architecture, competing microbial flora, chemically unique lumenal fluids, and different host defense properties. The ability to adapt to these various conditions appears to be inherent in the pathogen's ability to thrive in the host. The virulence factors described above do not represent an exhaustive list but portray some of the functions of the gonococcus that illustrate the principles involved in the modern understanding of virulence. Based on the example of *N. gonorrhoeae,* the nature of microbial virulence can be summarized in the following terms:

1. Pathogenicity may result from specific microbial virulence factors or activities that a) cause harm to host tissues or disrupt some physiologic process or b) inactivate or otherwise subvert host defenses.
2. Adherence to mucosal sites represents the first step in pathogenesis and is attributed to molecular structures on the microbial surface which bind to ligands on susceptible host tissues. In the gonococcus, in addition to the pili which serve as the major adhesins, there is evidence to support the ability of other surface glycolipids and Opa (opacity) proteins to bind to host cells as well.[10,11]
3. Regulation of gene function in response to the microenvironment is an essential aspect of virulence. In addition to causing the induction of 1 virulence attribute, genetic regulation may simultaneously affect several genetic loci. Such transcription of several different gene products in response to a single stimulus, described as global (coordinate) regulation, is becoming recognized as an important motif in virulence regulation. Microorganisms are able to utilize well-characterized sensory and control systems to induce the transcription of gene products that allow the organism to persist in the host. Numerous environmental signals have been identified in bacteria. In addition to iron, the gonococcus senses oxygen tension and nutrients in regulating the expression of some of its genes.
4. Invasion can be loosely defined as the ability of an organism to cross barriers in the host, resulting in the infectious process moving or spreading from its primary site. This process may take the form of microorganisms entering tissues either by interstitial spread to cross barriers (such as an epithelial surface) or it may represent the entry of the microorganism into cells. The term invasin has been applied to bacterial products that are specifically responsible for the entrance of microorganisms into tissues. Fallopian-tube explant culture has shown that the gonococcus is able to reach the basement membrane by entering a columnar epithelial cell at the apical surface, traveling through the cell, and exiting by exocytosis at the basal portion of the cell.[12] There is some evidence to indicate that the porin protein (outer-membrane protein P I) may play a role in coalescence with host membranes, ultimately leading to internalization of the gonococcus.[12] Although the role of P I is not yet certain, for purposes of the present discussion, it is useful to recognize that the gonococcus has some factor that functions as an invasin, whether or not the invasin is also a porin.
5. Intoxication, or the production of microbial products that cause some identifiable damage to host cells or interfere with cellular functions, is better known for pathogens other than *N. gonorrhoeae.* Despite this, most investigators believe that the toxic properties of the gonococcal lipooligosaccharide on the outer membrane of the organism and possibly the peptidoglycan of the cell wall contribute to some of the damage it produces in vivo. The ciliostasis and histopathology occurring in fallopian-tube explants exposed to the gonococcus are believed to be due to lipooligosaccharide.

After this review of some of the aspects of the gonococcus which account for its virulence, it is appropriate to consider whether virulence factors exist in microorganisms that are inconsistently found in pathogenic relationship with the host.

### PATHOGENICITY OF NORMAL FLORA ORGANISMS

Little space will be devoted here to the concept,[13] developed elsewhere, that the indigenous microbial flora of the vagina is a diverse collection of aerobic and anaerobic organisms living in a dynamic relationship with the colonized epithelium. Although colonization of the lower female genital tract is generally benign, many microorganisms are involved, from time to time, in symptomatic infections. Among the conditions for which the normal cervicovaginal flora has relevance are the polymicrobial infections involving operative sites after surgical procedures, postpartum endomyometritis, salpingeal and ovarian infections that occur in tempo with lower-tract gonococcal and/or chlamydial infections, and the vaginal conditions of mucocutaneous candidiasis and bacterial vaginosis.

Because the primary emphasis of this article is on the virulence of microorganisms, host defense will receive little attention here. Certainly, in the case of surgical infections, postpartum infections, and the mixed upper-tract invasion characteristic of pelvic inflammatory disease, a breach of the host defenses is of paramount importance. These represent cases in which significant numbers of normal flora organisms are precipitously transferred to an environment that may be conducive to rapid proliferation. It is tempting to attribute the symptoms of infection in these cases solely to the location of the organisms and not to any virulence properties they possess. This view is very likely a short-sighted assessment of the pathogenesis of these infections, although the research on virulence factors in these organisms is limited. A recent review by Brook[14] noted the importance of synergy between aerobic and anaerobic organisms coexisting at an infected site. Synergy in this setting may be attributed to various factors including the consumption of oxygen by aerobic species or production of antiphagocytic factors by anaerobic species. For example, succinate produced by some species of anaerobic bacteria has been shown to have an adverse effect on neutrophil chemotaxis.[15] Brook[14] also emphasized the abscessogenic property of anaerobic bacteria (*Bacteroides*, *Porphyromonas*, and *Prevotella* spp.) which may add to the virulence of the organisms through antiphagocytic activity. Finally, in view of the virulence enhancement that comes from including blood in bacterial inocula, the surgical site would seem to be a prime location for bacteria to undergo virulence induction. The possibility that organisms associated with vaginal symptoms, i.e., *Gardnerella*, anaerobic flora, *Candida*, also possess inducible virulence attributes should be considered. Among vaginal organisms, *C. albicans* has perhaps undergone the closest scrutiny in recent years because it is among the most important causes of nosocomial infections and is a common cause of mucocutaneous disease in acquired immunodeficiency syndrome (AIDS) patients. In the gynecologic context, the organism is of interest as a cause of vaginal symptoms, although it is also appropriately deemed to be a part of the host's normal flora and, in its role as natural flora, it would seem to be a low-virulence organism. In the vagina, it displays little more than microinvasiveness, but other virulence characteristics such as attachment, interaction with host defense factors, and toxigenicity exist. Because the organism can flourish in the vagina either as a commensal or as a cause of vaginal symptoms or can disseminate to cause septicemia with multiple organ infections, virulence regulation in this organism is probably a necessity.

### Attachment of Low-Virulence Organisms to Host Tissues

Attachment of the organism to the epithelial surface is a significant aspect of the life of a vaginal microorganism. Various studies of attachment have been reported which have provided some information about the mechanisms of fungal adherence, but some caution is warranted in evaluating these studies. Some investigations employed buccal epithelium as a surrogate for the vaginal epithelial cell. Although *Candida* adheres to both cell types, it is not clear whether the mechanism of adherence is identical for both cell types. The ability of *Candida* to enter a mycelial phase is an important consideration, and some evidence indicates that germinated cells adhere better to vaginal epithelium than do yeast cells.[16] We found that rats artificially inoculated with yeast-phase organisms only became colonized when they were well estrogenized and that, when viewed by scanning electron microscopy, the yeast inoculum had become uniformly mycelial.[17] Moreover, the electron micrographs suggested that these mycelial organisms showed superficial invasion of the epithelium, which one might view as an extreme form of attachment.

Heterogeneity among vaginal epithelial cells further complicates studies of microbial adhesion. In addition to individual variation in tissue architecture, vaginal epithelium undergoes different degrees of maturation at different times within a single individual. In our laboratory, we have used fluorescence microscopy to accurately quantitate various strains of yeast attached to exfoliated vaginal cells. By evaluating yeast adherence to vaginal cells from several donors against several yeast strains, we determined that patient-to-patient variation in epithelial-cell adhesiveness was greater than interstrain variation in yeast adhesiveness (Larsen, unpublished results, 1992). This result was anticipated since considerable variation in yeast adherence to buccal cells was noted by Sandin and coworkers.[18]

Future studies will need to determine the precise adhesive structures involved in yeast adherence to vaginal cells at various stages of maturation and whether different adhesins exist in yeast vs. mycelial forms of the organism. Although the mannoproteins of the yeast are thought to represent the major adhesins,[19,20] the ability of yeast to bind fibrinogen[21] has led to the concept that *Candida* may have ligand for fibronectin. Kalo et al.[22] suggested that fibronectin on intermediate cells of the female genital tract was a good ligand for *C. albicans*, but superficial cells had far less fibronectin and were therefore less receptive to yeast adherence. Vaginal epithelial maturation to the intermediate stage is characteristic of states of high circulating progesterone (as in pregnancy), so these observations may have at least a theoretical clinical correlation. Sawyer and colleagues[23] found that the RGD (arg-gly-asp) domain binds to *Candida* and opsonizes them for Kuppfer-cell phagocytosis and may have an important role in hepatic trapping of the organism. This property may have more relevance to disseminated infection than to mucocutaneous colonization. More to the issue of mucocutaneous candidiasis is the work of Ollert and coworkers,[24] who studied the inhibition of yeast adherence to keratinocytes in the presence of various inhibitors. While fibronectin provided some competition for yeast-keratinocyte interaction, RGD did not. Greater inhibition was achieved with saccharide inhibitors, most notably D-glucosamine. These authors also found the acid proteinase inhibitor pepstatin A to be an effective inhibitor of adherence in their assay system which they considered to provide evidence that secreted protease also plays a role in adhesion. The various observations on yeast attachment are consistent with the presence of major and minor adhesins that function in different environments. Work has shown that a *Candida* receptor analog which binds C3b participates in an integrin-like binding to RGD sequences and, in addition to anticomplement activity, may be important in fungal attachment to endothelial cells.[25]

Microbial attachment has been less studied for other organisms of relevance in the lower female genital tract. *G. vaginalis*, which is common among asymptomatic women and seen in abundance in individuals with bacterial vaginosis, is noted for its adherence to epithelial cells. Heavily colonized exfoliated vaginal cells have been described as clue cells, although the molecular mechanisms of adherence have not been worked out. Boustouller et al.[26] attributed the responsibility of attachment to vaginal epithelial cells to pili, whereas Scott and colleagues[27] considered an outer fibrillar matrix responsible for cytadhesion, with pili functioning as a hemagglutinin.

It must be emphasized that, despite the importance of microbial adherence in disease processes, attachment to tissue does not identify an organism as a pathogen. Indeed, vaginal lactobacilli possess adhesins which probably serve to promote their stable colonization of the vaginal epithelium, possibly with beneficial effect for the host.[28] Thus, whether the microbial species is highly virulent or only modestly virulent, its successful interaction with the host tissue depends on adhesive factors.

### Virulence Regulation in Low-Virulence Organisms

An additional characteristic of virulence is the ability to genetically regulate virulence attributes. The genetics of *C. albicans* is beginning to receive considerable research attention,[29] although studies on regulation of virulence factors remain rudimentary. It is clear that *Candida* can regulate some of its virulence factors, although details of how and why such regulation occurs are obscure. Furthermore, the regulation of virulence is currently recognized at the level of phenotype rather than at the level of genetic control. It will be sufficient at this point to mention a few of the systems that appear to undergo regulation in *Candida*.

The most obvious type of regulation involves the morphology of the organism. The ability of the organism to germinate has long been associated with its pathogenic function. This concept is based on the fact that the usual form of the fungus in the host is the mycelial phase. This form is considered to be too cumbersome to be readily phagocytosed and is believed to possess microinvasive properties. This may be reflected in the ability of hyphal organisms to bind fibrinogen, laminin, and complement products, a property not shared by the yeast form.[30–32] It is well known that placing yeast cells of *C. albicans* in serum causes their prompt germination. In addition, the recent report of DeBernardis and coworkers[33] suggests that the contact of yeast with epithelium triggers a conversion to mycelial growth including the expression of new surface mannoprotein epitopes. Thus, the organism displays the ability to sense its environment and respond to it in a manner that theoretically enhances its virulence.

The discovery of a complement receptor analog on *C. albicans* and *C. stellatoidea* by Heidenreich and Dierich[34] revealed an important potential virulence attribute of the organism. It is postulated that this receptor analog serves as an antiphagocytic factor by binding complement split products which normally bind to neutrophil receptors.[35] It is notable that these investigators found this factor associated with hyphal forms of the organism[35] and demonstrated that it was induced by elevated glucose concentrations.[36] To describe these events as a globally regulated virulence system goes beyond current knowledge and will require confirming basic genetic investigation. Even so, this system appears to function as a regulated virulence system.

*C. albicans* also appears to spontaneously undergo a phenotypic change in colonial morphology that has been described as "high-frequency switching."[37] The spontaneous changes that occur in colony type are reminiscent of the spontaneous changes and gonococcal antigen presentation described earlier. Switching probably involves genetic mechanisms different from those seen in the gonococcus, but a similar purpose of evading the immune system may accrue to the switching *Candida*.[38]

One important characteristic of global regulation is a sensor mechanism or a chemoreceptor that identifies the changing environment for the microorganism. The effects of various nutrients in relation to adherence phenomena were reviewed by Calderone and Braun[19] and are not repeated here. An alternative sensory mechanism that may have particular significance for *Candida* in the mammalian host is estrogen-binding activity.[39] Transcriptional regulating activity similar to that of classic mammalian estrogen receptors has not been observed in yeast, but there are discernible biological effects of estrogen on *C. albicans*. In our laboratory, we have noted that micromolar estradiol increases biomass production in vitro and enhances the production of toxin.[40]

### Toxigenicity of Low-Virulence Organisms

Clearly, the organisms that normally associate with the host as normal commensal flora have been given little attention as toxin producers. But the production of toxic substances with subtle effects on the host physiology or immunity could play a role in the virulence of these organisms. As noted for the gonococcus, the endotoxin it produces may not serve as its primary virulence factor, but is probably responsible for some of its effects in the upper genital tract or in disseminated disease.

*G. vaginalis* produces a hemolysin which is one of its key identifying characteristics. Research from our laboratory[41–43] and that of other investigators[44,45] have characterized the hemolytic toxin from this organism. There is ample precedent among other bacteria to indicate that hemolysins contribute to microbial virulence. Despite the fact that *Gardnerella* alone is not usually involved in infections, its hemolysin may contribute to this organism's participation in bacterial vaginosis.

We found that the hemolysin is produced in soluble form in the presence of serum or other releasing factors, but in serum-free medium the hemolysin remains bound to the bacterial cell.[41] Biochemical evidence indicates that this is a protein toxin which may be stabilized by associated lipid[42] and its native form is probably an aggregate of 60–65 KD monomers.[41] Although this toxin lyses human erythrocytes, its action against polymorphonuclear neutrophils may be more clinically relevant.[43]

The paucity of leukocytes in cases of bacterial vaginosis is well known. We found that as few as 2 hemolytic units/ml of *G. vaginalis* hemolysin was able to arrest the chemotaxis of human neutrophils.[43] In anaerobic culture in peptone-yeast extract-glucose medium with serum, the organism typically produced several hundred hemolytic units/ml. The microbial ecology of bacterial vaginosis is undeniably complex, and no single bacterium or bacterial factor is responsible for the clinical picture. However, the observed biological activities of the *G. vaginalis* hemolysin suggest its participation in this condition.

Another organism that is not usually considered to be toxigenic is *C. albicans*. As mentioned above, *C. albicans* interacts with the host in some very complex ways, but, despite the many investigations on this organism, few studies consider the possibility of exotoxin production. Based on sporadic claims in the literature that *Candida* has immunosuppressive properties, we began looking for an immunosuppressive mycotoxin. Some filamentous fungi produce epipolythiodioxopiperazines that have immunosuppressive and antiphagocytic activity. We developed a chromatographic procedure for the best-known compound of this class of toxins and determined that gliotoxin was present in the spent culture supernatants of *C. albicans*.[46] We verified the structure of the compound with mass spectroscopy.[40] Although the compound exerts various adverse effects on neutrophil function including an inhibition of directed migration (Shah and Larsen, unpublished observations), it also has some interesting stimulatory functions on isolated neutrophils. Gliotoxin may cause degranulation and premature release of lactoferrin from neutrophils and enhance respiratory burst activity at very low concentrations. The potential for these actions to influence the development of mucocutaneous disease is clear, but additional work is needed to define the role of this immunomodulating toxin. One clear aspect of gliotoxin production is that estradiol provides a stimulus for toxin production.[40] As previously emphasized, this finding suggests that toxin production, along with various other virulence attributes in *C. albicans*, may be globally regulated.

### CONCLUSIONS

Contemporary investigation of the pathogenic mechanisms of infectious agents has revealed a broad range of virulence attributes that damage host cells or interfere with host functions. Moreover, these virulence attributes are often globally regulated in response to environmental signals. Little attention has been given to low-virulence organisms associated with the host as part of the normal flora. In this article, *N. gonorrhoeae*, was selected to illustrate virulence characteristics that include 1) interaction with host defenses, 2) adhesion to host tissues, 3) regulation of virulence attributes, and 4) elaboration of toxins. Evidence is presented to show that similar virulence properties are associated with the microbial inhabitants of the female lower genital tract. Far from being irrelevant to low-virulence organisms, the study of virulence factors is a fruitful area of investigation and one that may positively influence the health of women.

### REFERENCES

1. Cornelium GR: Yersiniae, finely tuned pathogens. In Hormaeche CE, Penn CW, Smyth CJ (eds): Molecular Biology of Bacterial Infection, Current Status and Future Perspectives. Cambridge: Cambridge University Press, pp 231–266, 1992.
2. Smith T: Parasitism and Disease. Princeton: Princeton University Press, 1934.
3. Robinson BD, Meyer TF: Antigenic variation in bacterial pathogens. In Hormaeche CE, Penn CW, Smyth CJ (eds): Molecular Biology of Bacterial Infection, Current Status and Future Perspectives. Cambridge: Cambridge University Press, pp 61–73, 1992.
4. Meyer TF, Gibbs CP, Haas R: Variation and control of protein expression in *Neisseria*. Annu Rev Microbiol 44: 451–477, 1990.
5. Kornfeld SJ, Plaut AG: Secretory immunity and the bacterial IgA proteases. Rev Infect Dis 3:521–534, 1981.
6. Pohlner J, Halter R, Beyreuther K, Meyer TF: Gene structure and extracellular secretion of *Neisseria gonorrhoeae* IgA protease. Nature 325:458–462, 1987.
7. Weinberg ED: Roles of iron in host-parasite interactions. J Infect Dis 124:401–410, 1971.
8. Blanton KJ, Biswas GD, Tsai J, Adams J, Dyer DW, Davis SM, Koch GG, Sen PK, Sparling FP: Genetic evidence that *Neisseria gonorrhoeae* produces specific receptors for transferrin and lactoferrin. J Bacteriol 172: 5225–5235, 1990.
9. Stevenson P, Williams P, Griffiths E: Common antigenic domains in transferrin-binding protein 2 of

*Neisseria meningitidis, Neisseria gonorrhoeae* and *Hemophilus influenzae* type b. Infect Immun 60:2391–2396, 1992.

10. Beeson D, Gotschlich EC: Interactions of gonococci with HeLa cells: Attachment and the role of protein II. Infect Immun 54:154–160, 1986.
11. Paruchuri DK, Seifert HS, Ajioka RS, Karisson KA, So M: Identification and characterization of a *Neisseria gonorrhoeae* gene encoding a glycolipid-binding adhesin. Proc Natl Acad Sci USA 87:333–337, 1990.
12. McGee Z: Pathogenic mechanisms of *Neisseria gonorrhoeae:* Observations on damage to human fallopian tubes in organ culture by gonococci of colony type 1 or 4. J Infect Dis 143:413, 1981.
13. Larsen B: Microbiology of the female genital tract. In Pastorek JG II (ed): Obstetric and Gynecologic Infectious Disease. New York: Raven Press, pp 11–25, 1994.
14. Brook I: Concepts of mixed aerobic-anaerobic obstetric and gynecologic infections. In Pastorek JG II (ed): Obstetric and Gynecologic Infectious Disease. New York: Raven Press, pp 37–46, 1994.
15. Eftimiadi C, Buzzi E, Tonetti M, Buffa P, Buffa D, VanStenbergen JTJ, deGraaff J, Botta GA: Short chain fatty acids produced by anaerobic bacteria alter the physiologic responses of human neutrophils to chemotactic peptide. J Infect 14:43, 1987.
16. Sobel JD, Muller G, Myers PG, Kaye D, Levison ME: *Candida albicans* adherence to vaginal epithelial cells. J Infect Dis 143:76–82, 1981.
17. Laren B, Galask RP: Influence of estrogen and normal flora on vaginal candidiasis in the rat. J Reprod Med 29:863–868, 1984.
18. Sandin RL, Rogers AL, Beneke ES, Fernandez MI: Influence of mucosal cell origin on the in vitro adherence of *Candida albicans:* Are mucosal cells from different sources equivalent? Mycopathologia 98:111–119, 1987.
19. Calderone RA, Braun PC: Adherence and receptor relationships of *Candida albicans.* Microbiol Rev 55:1–20, 1991.
20. Douglas LJ: Mannoprotein adhesins of *Candida albicans.* In Bennett JE, Hay RJ, Peterson PK (eds): New strategies in fungal disease (Frontiers of Infectious Diseases Series), Edinburgh: Churchill Livingstone, pp 34–50, 1992.
21. Bouali A, Robert R, Tronchin G, Senet J-M: Characterization of binding of human fibrinogen to the surface of germ-tubes and mycelium of *Candida albicans.* J Gen Microbiol 133:545–551, 1987.
22. Kalo A, Segal E, Sahar E, Dayan D: Interaction of *Candida albicans* with genital mucosal surfaces: Involvement of fibronectin in adherence. J Infect Dis 157:1253–1256, 1988.
23. Sawyer RT, Garner RE, Judson JA: Arg-gly-asp (RGD) peptides alter hepatic killing of *Candida albicans* in the isolated perfused mouse liver model. Infect Immun 60: 213–218, 1992.
24. Ollert MW, Sohnchen R, Korting HC, Ollert U, Brautigam S, Brautigam W: Mechanisms of adherence of *Candida albicans* to cultured human keratinocytes. Infect Immun 61:4560–4568, 1993.
25. Gustafson KS, Vercellotti GM, Bendel CM, Hostetter MK: Molecular mimicry in *Candida albicans:* Role of an integrin analogue in adhesion of the yeast to human endothelium. J Clin Invest 87:1896–1902, 1991.
26. Boustouller YL, Johnson AP, Taylor-Robinson D: Pili on *Gardnerella vaginalis* studied by electron microscopy. J Med Microbiol 23:327–329, 1987.
27. Scott TG, Curran B, Smyth CJ: Electron microscopy of adhesive interactions between *Gardnerella vaginalis* and vaginal epithelial cells, McCoy cells and human red blood cells. J Gen Microbiol 135:475–480, 1989.
28. Nagy E, Froman G, Mardh P-A: Fibronectin binding of *Lactobacillus* species isolated from women with and without bacterial vaginosis. J Med Microbiol 37:38–42, 1992.
29. Kurtz MB, Kirsch DR, Kelly R: The molecular genetics of *Candida albicans.* Microbiol Sci 5:58–63, 1988.
30. Bouchara J, Tronchin G, Annaix V, Robert R, Senet JM: Presence of laminin receptors on *Candida albicans* germ tubes. Infect Immun 58:48–54, 1990.
31. Calderone RA, Lineham NL, Wadsworth E, Sandberg AL: Identification of C3d receptors on *Candida albicans.* Infect Immun 56:252–258, 1988.
32. Tronchin G, Robert R, Douali A, Senet JM: Immunocytochemical localization of in vitro binding of human fibrinogen to *Candida albicans* germ tube and mycelium. Ann Inst Pasteur (Microbiol) 138:177–187, 1987.
33. DeBernardis F, Moilinari A, Boccanera M, Stringaro A, Robert R, Senet J-M, Arancia G, Cassone A: Modulation of cell surface-associated mannoprotein antigen expression in experimental *Candida* vaginitis. Infect Immun 62:509–519, 1994.
34. Heidenreich F, Dierich MP: *Candida albicans* and *Candida stellatoidea,* in contrast to other *Candida* species, bind iC3b and C3d but not C3b. Infect Immun 50:598–600, 1985.
35. Gilmore BJ, Retsinas EM, Lorenz JS, Hostetter MK: An iC3b receptor on *Candida albicans:* Structure, function and correlates for pathogenicity. J Infect Dis 157:38–46, 1988.
36. Hostetter MK, Lorenz JS, Preus L, Kendrick KE: The iC3b receptor on *Candida albicans:* Subcellular localization and modulation of receptor expression by glucose. J Infect Dis 161:761–768, 1990.
37. Slutsky B, Boffo J, Soll DR: High frequency "switching" of colony morphology in *Candida albicans.* Science 230: 666–669, 1985.
38. Soll DR, Galask RP, Isley S, Rao TVG, Stone D, Hicks J, Schmid J, Mac K, Hanna C: Switching of *Candida albicans* during successive episodes of recurrent vaginitis. J Clin Microbiol 27:681–690, 1989.
39. Skowronski R, Feldman D: Characterization of an estrogen-binding protein in the yeast *Candida albicans.* Endocrinology 124:1965–1972, 1989.
40. Shah DT, Larsen B: Identity of a *Candida albicans* toxin and its production in vaginal secretions. Med Sci Res 20:353–355, 1992.
41. Shubair M, Snyder IS, Larsen B: *Gardnerella vaginalis*

hemolysin. I. Production and purification. Immunol Infect Dis 3:135–142, 1993.
42. Shubair M, Snyder IS, Larsen B: *Gardnerella vaginalis* hemolysin. II. Kinetics of cell lysis and relation to other hemolysins. Immunol Infect Dis 3:143–148, 1993.
43. Shubair M, Snyder IS, Larsen B: *Gardnerella vaginalis* hemolysin. III. Effects on human leukocytes. Immunol Infect Dis 3:149–153, 1993.
44. Rottini G, Dobrina A, Forgiarini O, Nardon E, Amirante GA, Patriarca P: Identification and partial characterization of a cytolytic toxin produced by *Gardnerella vaginalis*. Infect Immun 58:3751–3758, 1990.
45. Kretschmar UM, Hammann R, Kutzner HJ: Purification and characterization of *Gardnerella vaginalis* hemolysin. Curr Microbiol 23:7–13, 1991.
46. Shah DT, Larsen B: Clinical isolates of yeast produce a gliotoxin-like substance. Mycopathologia 116:203–208, 1991.
